# The Age-Modified Shock Index: Predicting Massive Transfusion and Mortality in Traumatic Injury Patients

**DOI:** 10.1155/emmi/8754824

**Published:** 2025-06-04

**Authors:** Soo Bin Choi, Suck Ju Cho, Seok-Ran Yeom, Sung-Wook Park, Young Mo Cho, Up Huh, Yeaeun Kim, Dongman Ryu, Chanhee Song, Won Ung Tae, Il Jae Wang

**Affiliations:** ^1^Department of Emergency Medicine, Pusan National University School of Medicine and Biomedical Research Institute, Pusan National University Hospital, Busan, Republic of Korea; ^2^Department of Thoracic and Cardiovascular Surgery, Pusan National University School of Medicine and Biomedical Research Institute, Pusan National University Hospital, Busan, Republic of Korea; ^3^Department of Health Care Management, Catholic University of Pusan, Busan, Republic of Korea; ^4^Medical Research Institute, Pusan National University, Busan, Republic of Korea

**Keywords:** age shock index, injury, massive transfusion, shock index

## Abstract

**Background and Purpose:** Previous studies have demonstrated that the shock index (SI), age-adjusted shock index (ASI), and modified shock index (MSI) are useful for predicting massive transfusion (MT) and mortality in patients with traumatic injuries. However, studies have not been conducted on the use of the age-modified shock index (AMSI) to indicate the prognosis of patients with traumatic injuries. This study aimed to evaluate the predictive power of AMSI for MT and mortality. We hypothesized that AMSI would be superior to other indices in predicting outcomes in patients with traumatic injuries.

**Methods:** This retrospective, single-center study was conducted at a level 1 trauma center and included consecutive patients who visited the trauma center between January 2016 and December 2022. The predictive value of AMSI for MT, in-hospital mortality, and 24 h mortality was assessed using receiver operating characteristic (ROC) analysis. We compared the area under the ROC curve (AUROC) of AMSI with those of SI, ASI, and MSI.

**Results:** In total, 6591 patients were included in the study, of whom 479 received MT. The in-hospital and 24 h mortality rates were 8.7% and 5.3%, respectively. The SI, ASI, MSI, and AMSI all showed better predictive performance for MT (AUC > 0.7) than that for in-hospital (AUC: 0.50, 0.61, 0.50, and 0.62) and 24 h mortality (AUC: 0.54, 0.56, 0.54, and 0.56). However, AMSI did not demonstrate superior performance compared with the other indices (SI, ASI, and MSI) in predicting both MT and 24 h mortality. AMSI demonstrated significantly better predictive performance for in-hospital mortality than the other indices; however, the difference from ASI was not substantial. This is likely because age has a significant impact on in-hospital mortality.

**Conclusion:** Indices other than AMSI that are easier to compute may be more useful for the prognostic evaluation of patients with traumatic injuries.

## 1. Introduction

Trauma remains a leading cause of death worldwide and poses a significant global health challenge. Massive hemorrhage is one of the primary causes of mortality in patients [1, 2]. Thus, accurately predicting the prognosis of patients with traumatic injuries is crucial for improving outcomes and guiding appropriate interventions such as massive transfusion (MT) [3–7].

The shock index (SI), introduced by Allgower and Burri, is simple and convenient to use and represents the ratio of heart rate (HR) to systolic blood pressure (SBP) [8]. It is widely used in trauma assessment and helps to predict the severity and prognosis of trauma [9, 10]. Efforts have been made to enhance its predictive capability by adding other important parameters such as the Glasgow Coma Scale (GCS) score, age, and diastolic blood pressure (DBP). These modified indices have shown better predictive values than those shown by SI [11–13].

The age-modified shock index (AMSI) was first introduced in 2020 by Zhou et al. as a predictor of mortality in patients with acute myocardial infarction [14]. Subsequent studies evaluated the prognostic accuracy of the AMSI, which integrates DBP and age with SI, in predicting mortality in patients with conditions such as acute myocardial infarction, acute stroke, and heart failure [14–18]. However, no studies have been conducted on the use of AMSI in patients with traumatic injuries.

In this study, we aimed to evaluate the predictive ability of AMSI for MT, in-hospital mortality, and 24 h mortality in patients with traumatic injuries. In addition, we compared the predictive performance of AMSI with those of SI, age-adjusted shock index (ASI), and modified shock index (MSI). We hypothesized that AMSI would serve as a useful predictor in patients with traumatic injuries and demonstrate superior predictive ability compared with that of other indices.

## 2. Materials and Methods

### 2.1. Study Design and Population

This retrospective single-center study was conducted at the Pusan National University Hospital (PNUH) Trauma Center. The PNUH Trauma Center functions as a major level 1 trauma center for the Pusan and Gyeongnam regions, which have a population of 7.7 million people, is one of the largest trauma centers in South Korea. Approximately 1000 severely injured patients with traumatic injuries and an injury severity score (ISS) of > 15 are treated annually at the PNUH Trauma Center.

This study enrolled patients from the independent trauma center emergency department (ED) at PNUH, which is separate from the general ED. The admission criteria for the trauma center ED are based on the “Guidelines for field triage of injured patients—steps one and two” [19], but admission is ultimately determined by the emergency physician. If the patient did not meet the field triage criteria, but the emergency physician's judgment indicated severe trauma, the patient was admitted to the trauma center ED.

This study included 8129 patients who presented to the trauma center between January 2016 and December 2022. The exclusion criteria were as follows: (1) age < 16 years; (2) cardiac arrest upon arrival at the trauma center; and (3) missing data for age, SBP, DBP, and HR.

This study was approved by the Institutional Review Board of PNUH (IRB approval number: 2405-018-139). The requirement for informed consent was waived because the research data were anonymized and retrospectively analyzed.

### 2.2. Data Collection and Outcome Measures

Research data were obtained in the form of electronic medical records (EMRs) from the computerized PNUH system, and personal information was removed from the research data in advance to ensure anonymity. The following information was extracted: age, sex, mechanism of injury, vital signs (SBP, DBP, and HR) at arrival, GCS score at arrival, ISS, MT, in-hospital mortality, and 24 h mortality. In addition, the following laboratory data, obtained during trauma center visits, were collected: prothrombin time international normalized ratio (PT INR), activated partial thromboplastin time (aPTT), hemoglobin level, and platelet count.

SI, ASI, MSI, and AMSI were calculated using the following formulas:

(1) $$\begin{array}{c} \text{SI}=\frac{\text{HR}}{\text{SBP}}, \end{array}$$

(2) $$\begin{array}{c} \text{ASI}=\text{age}\times \left(\frac{\text{HR}}{\text{SBP}}\right), \end{array}$$

(3) $$\begin{array}{c} \text{MSI}=\frac{\text{HR}}{\text{mean arterial pressure }\left(\text{MAP}\right)}, \end{array}$$

(4) $$\begin{array}{c} \text{AMSI}=\text{age}\times \left(\frac{\text{HR}}{\text{MAP}}\right). \end{array}$$

The primary outcome was MT. We defined MT as follows: (a) transfusion of more than 10 units of packed red blood cells (pRBCs) within 24 h or (b) transfusion of more than 5 units of pRBCs within 4 h of visiting the trauma center [20–22]. The secondary outcomes were in-hospital and 24 h mortality.

### 2.3. Statistical Analyses

Continuous variables were assessed for normality using the Kolmogorov–Smirnov test. Variables following a normal distribution were presented as mean ± standard deviation (SD) and compared between the two groups using the independent *t*-test, while non-normally distributed variables were presented as median and interquartile range (IQR) and analyzed using the Mann–Whitney *U* test.

To evaluate the predictive power of SI, ASI, MSI, and AMSI for MT, in-hospital mortality, and 24 h mortality in patients with severe traumatic injuries, we performed receiver operating characteristic (ROC) curve analysis and calculated the area under the curve (AUC). The areas under the ROC curves (AUROCs) were then compared using DeLong's method.

Statistical analyses were performed using MedCalc Version 22.007 for Windows. All tests were two-tailed, and *p* values < 0.05 were considered statistically significant.

## 3. Results

### 3.1. Patients' Characteristics

**Figure 1:**
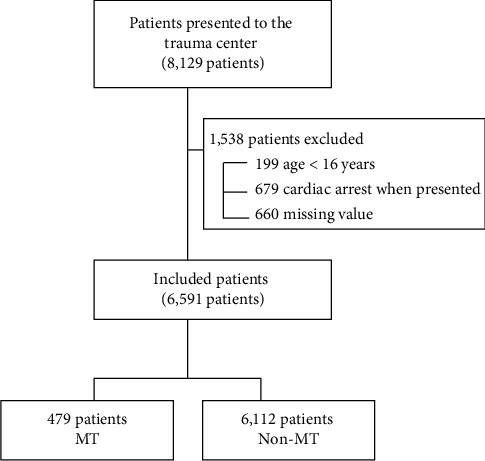
Study flow diagram. MT, massive transfusion.

Between January 2016 and December 2022, 8129 patients visited the trauma center. A total of 1538 patients were excluded from the study based on the exclusion criteria. The exclusion criteria were as follows: age < 16 years (*n* = 199), cardiac arrest upon presentation to the ED (*n* = 679), and missing data (*n* = 660). Thus, 6591 patients were included in this study (Figure 1).

Among all patients, 5050 (76.6%) were men and 1541 (23.4%) were women, with men outnumbering women. The median age was 60 years (IQR: 70.00, 45.00), and the median ISS was 17.00 (IQR: 26.00, 10.00). The most common mechanism of injury was traffic collisions, with 3213 cases (48.8%). The in-hospital and 24 h mortality rates were 575 (8.7%) and 352 (5.3%), respectively. MT was administered to 479 (7.3%) patients.

### 3.2. Comparison Between MT and Non-MT Groups

We compared data for the group that received MT with those for the group that did not receive MT (Table 1). The median age and IQR were 60.00 (74.00, 48.00) in the MT group and 60.00 (70.00, 45.00) in the non-MT group. The difference was statistically significant, with a *p* value of 0.0008. No significant sex-related differences were observed between the MT and non-MT groups. The proportion of trauma mechanisms involving traffic collisions in the MT group was significantly higher at 273 (57%) compared with 2940 (48.1%) in the non-MT group. In the MT group, SBP (*p* < 0.0001), DBP (*p* < 0.0001), and GCS (*p* < 0.0001) were significantly lower than those in the non-MT group whereas HR (*p* < 0.0001) was significantly higher in the MT group than those in the non-MT group.

The median ISS and IQR were 29.00 (38.00, 22.00) in the MT group, significantly higher than 17.00 (25.00, 10.00) in the non-MT group. ISS was categorized into three groups: minor (ISS 1–8), moderate (ISS 9–15), and severe (ISS ≥ 16) [23, 24]. In the MT group, 441 cases (92.1%) had an ISS of 16 or higher, a significantly higher proportion than 3683 cases (60.3%) in the non-MT group. Conversely, in the minor ISS group (ISS 1–8), only 8 cases (1.7%) were observed in the MT group, which was significantly lower than 912 cases (14.9%) in the non-MT group.

Laboratory test results showed that in the group receiving MT, PT INR (*p* < 0.0001) and aPTT (*p* < 0.0001) were significantly higher than those in the non-MT group whereas Hb (*p* < 0.0001) and platelet (*p* < 0.0001) levels were significantly lower in the MT group than those in the non-MT group. In addition, in the group that received MT, the in-hospital (*p* < 0.0001) and 24 h mortality rates (*p* < 0.0001) were significantly higher than those in the group that did not receive MT.

### 3.3. ROC Analysis

**Figure 2:**
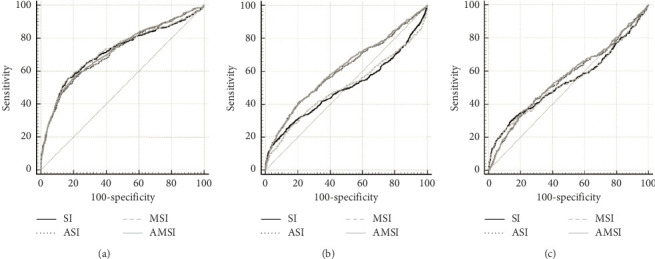
Receiver operating characteristic curve for (a) MT, (b) in-hospital mortality, and (c) 24 h mortality.

To evaluate the predictive ability of SI, ASI, MSI, and AMSI for MT, in-hospital mortality, and 24 h mortality, we conducted ROC analysis and calculated the AUROCs (Figure 2 and Table 2). The SI, ASI, MSI, and AMSI all showed better predictive performance for MT (AUC > 0.7) than that for in-hospital and 24 h mortality. For the prediction of MT, the AUROC values of SI, ASI, MSI, and AMSI were all approximately 0.72. To predict in-hospital mortality, the AUROC values for SI, ASI, MSI, and AMSI were 0.50, 0.61, 0.50, and 0.62, respectively. For predicting 24 h mortality, SI, ASI, MSI, and AMSI had AUROC values of 0.54, 0.56, 0.54, and 0.56, respectively.

## 4. Discussion

In this study, we evaluated the predictive ability of SI, ASI, MSI, and AMSI for MT, in-hospital mortality, and 24 h mortality in patients with traumatic injuries. The ROC analysis demonstrated superior predictive performance during MT (AUCs, SI: 0.72, ASI: 0.72, MSI: 0.72, and AMSI: 0.72) compared with in-hospital mortality (AUCs, SI: 0.50, ASI: 0.61, MSI: 0.50, and AMSI: 0.62) and 24 h mortality (AUCs, SI: 0.54, ASI: 0.56, MSI: 0.54, and AMSI: 0.56). However, AMSI did not consistently demonstrate superior predictive performance for trauma patients compared to other indices.

The SI is a simple and convenient index, which represents the ratio of HR to SBP. It is easily calculated at the bedside during trauma assessment and has been widely used as a tool for evaluating hemodynamic instability in patients with traumatic injuries [8–10]. A high posttrauma SI value predicts a high risk of mortality and is useful for identifying major hemorrhages [8].

The SI has limitations because it excludes the influence of DBP and does not account for variables such as patient age. Other indices have been developed to address these limitations. Liu et al. reported that DBP is important for predicting the severity of trauma in patients in emergency situations. They recommended the inclusion of DBP in the SI to assess these patients and introduced MSI, which incorporates DBP in the form of MAP into SI [20]. MSI has shown superior mortality prediction ability compared with that shown by SI, allowing for a more accurate assessment of a patient's condition in acute situations such as hemorrhage [25, 26]. In addition, the SI value tends to decrease with age owing to the increase in SBP with advancing age [27]. To address this problem and improve predictive ability, ASI was introduced, in which the SI value is multiplied by age [15]. Neumann et al. demonstrated that ASI has a superior predictive ability for in-hospital mortality in elderly patients with traumatic injuries compared with that shown by SI and MSI [28]. Moreover, Kim et al. found an association between increased ASI and mortality in elderly patients with traumatic injuries in the ED [29]. These studies highlight the utility of SI, ASI, and MSI as prognostic indicators for patients with traumatic injuries.

AMSI, a new index derived from SI, integrates multiple parameters, including age, HR, SBP, and DBP [18]. It was first introduced in 2020 by Zhou et al. as a predictor of mortality in patients with acute myocardial infarction [14]. Subsequent studies have shown that AMSI effectively predicts mortality in conditions such as acute myocardial infarction, stroke, and heart failure [14–18]. Although SI has been extensively used for patients with traumatic injuries, no research has been conducted on the use of AMSI in these patients. To the best of our knowledge, this is the first study to evaluate the association between AMSI and prognosis in patients with traumatic injuries.

Contrary to our hypothesis, AMSI did not demonstrate superior performance compared to the other indices (SI, ASI, and MSI) in predicting both MT and 24 h mortality. No statistically significant evidence was found to support its superiority over SI, ASI, or MSI in these outcomes (Table 3). An exception was observed in in-hospital mortality, where AMSI showed statistically significantly better predictive performance than SI, ASI, and MSI. However, given the small AUC differences, its clinical significance in trauma patients remains uncertain. The superior predictive performance of AMSI in in-hospital mortality may be attributed to the significant impact of age on in-hospital mortality. Similarly, the fact that ASI, which also includes age as a variable, outperforms SI and MSI supports this assumption.

However, considering the findings of previous studies, it is not surprising that AMSI is not superior to the other indices. Bondariyan et al. found that although SI and its derivatives are useful in predicting in-hospital mortality in patients with acute heart failure, the predictive abilities of SI, ASI, MSI, and AMSI are similar [15]. Demir and Eren indicated that although AMSI is useful for predicting in-hospital mortality in patients with acute ischemic stroke, it is not significantly superior to ASI [16]. Wang et al. observed that AMSI outperformed SI and MSI in predicting complications after percutaneous coronary intervention in patients with ST-segment elevation myocardial infarction; however, the predictive ability of AMSI was similar to that of ASI [30].

Although the results of this single-center retrospective study cannot be generalized, they suggest that the use of AMSI, which is relatively more complex than other indices, is not essential for predicting MT and mortality in patients with traumatic injuries. AMSI demonstrated better predictive performance in in-hospital mortality. However, given that the AUC difference from ASI was not substantial, it is difficult to consider AMSI more useful, especially since it is more complex to calculate. The reasons why AMSI is not superior to the other indices are unclear. One possible explanation is the nonlinear physiological changes in blood pressure and HR with age. Another possibility is the difference in dynamic responses of blood pressure and HR between patients with conditions such as hypertension, diabetes, coronary artery disease, and medication use and healthy patients [9].

This study had several limitations. First, it was conducted at a single center based on retrospective observational data. Second, information on previous medication use, such as beta-blockers, antihypertensives, analgesics, or anxiolytics, was lacking, which could potentially influence SBP and HR, and thus affect the SI, ASI, MSI, and AMSI values.

## 5. Conclusions

AMSI does not outperform SI, ASI, and MSI in predicting MT and 24 h mortality in patients with traumatic injuries. Although it demonstrated better predictive performance in in-hospital mortality, the difference from ASI was not substantial. Therefore, indices other than AMSI that are easier to compute may be more useful for the prognostic evaluation of patients with traumatic injuries.

## Figures and Tables

**Table 1:** Characteristics of included patients.

Variables	Total (*n* = 6591)	MT (*n* = 479)	Non-MT (*n* = 6112)	*p* value
Age (y), median (IQR)	60.00 (70.00, 45.00)	60.00 (74.00, 48.00)	60.00 (70.00, 45.00)	*p* = 0.0008
Sex, *n* (%)				*p* = 0.3694
Male	5050 (76.62)	359 (74.95)	4691 (76.75)	
Female	1541 (23.38)	120 (25.05)	1421 (23.25)	
Mechanism of injury, *n* (%)				*p* < 0.0001
Traffic collisions	3213 (48.75)	273 (56.99)	2940 (48.10)	
Fall from height	1828 (27.73)	122 (25.47)	1706 (27.91)	
Ground level fall	437 (6.63)	7 (1.46)	430 (7.04)	
Object blunt	519 (7.88)	35 (7.31)	484 (7.92)	
Penetrating	425 (6.45)	33 (6.89)	392 (6.41)	
Etc	169 (2.56)	9 (1.88)	160 (2.62)	
SBP, median (IQR)	120.00 (140.00, 100.00)	90.00 (120.00, 80.00)	120.00 (140.00, 100.00)	*p* < 0.0001
DBP, median (IQR)	80.00 (90.00, 60.00)	60.00 (80.00, 40.00)	80.00 (90.00, 60.00)	*p* < 0.0001
HR, median (IQR)	88.00 (102.00, 76.00)	102.00 (120.00, 83.00)	88.00 (101.00, 76.00)	*p* < 0.0001
GCS at ED, median (IQR)	15.00 (15.00, 13.00)	9.00 (15.00, 5.00)	15.00 (15.00, 13.00)	*p* < 0.0001
ISS, median (IQR)	17.00 (26.00, 10.00)	29.00 (38.00, 22.00)	17.00 (25.00, 10.00)	*p* < 0.0001
Minor (ISS 1–8), *n* (%)	920 (13.96)	8 (1.67)	912 (14.92)	
Moderate (ISS 9–15), *n* (%)	1547 (23.47)	30 (6.26)	1517 (24.82)	
Severe (ISS ≥ 16), *n* (%)	4124 (62.57)	441 (92.07)	3683 (60.26)	
In-hospital mortality, *n* (%)				*p* < 0.0001
No	6016 (91.28)	276 (57.62)	5740 (93.91)	
Yes	575 (8.72)	203 (42.38)	372 (6.09)	
24-h mortality, *n* (%)				*p* < 0.0001
No	6239 (94.66)	375 (78.29)	5864 (95.94)	
Yes	352 (5.34)	104 (21.71)	248 (4.06)	
PT INR, median (IQR)	1.06 (1.15, 1.00)	1.23 (1.41, 1.11)	1.05 (1.13, 0.99)	*p* < 0.0001
aPTT time, median (IQR)	25.90 (29.40, 23.60)	29.70 (39.25, 25.50)	25.70 (28.90, 23.50)	*p* < 0.0001
Hemoglobin, median (IQR)	13.60 (15.00, 11.90)	11.60 (13.60, 9.20)	13.60 (15.30, 11.90)	*p* < 0.0001
Platelet, median (IQR)	221.00 (266.00, 176.00)	189.50 (244.00, 145.00)	223.00 (268.00, 179.00)	*p* < 0.0001

Abbreviations: aPTT, activated partial thromboplastin time; DBP, diastolic blood pressure; ED, emergency department; GCS, Glasglow coma scale; HR, heart rate; IQR, interquartile range; ISS, injury severity score; MT, massive transfusion; PT INR, prothrombin time international normalized ratio; SBP, systolic blood pressure.

**Table 2:** Predictive power of the SI, ASI, MSI, and AMSI for MT, in-hospital mortality, and 24 h mortality.

	AUC (95% CI)	Cut-off point	Sensitivity	Specificity	PPV	NPV
*MT*						
SI	0.72 (0.71–0.73)	0.99	55.74	82.98	20.1	96.0
ASI	0.72 (0.71–0.73)	53.95	56.99	78.16	17.0	95.9
MSI	0.72 (0.71–0.74)	1.39	53.86	85.05	21.9	95.9
AMSI	0.72 (0.71–0.73)	72.34	58.66	77.63	17.0	96.0

*In-hospital mortality*						
SI	0.50 (0.49–0.51)	0.53	26.78	85.26	14.8	92.4
ASI	0.61 (0.60–0.62)	54.86	41.91	78.22	15.5	93.4
MSI	0.50 (0.49–0.52)	0.67	77.91	10.85	7.7	83.7
AMSI	0.62 (0.61–0.63)	75.71	40.87	80.02	16.3	93.4

*24 h mortality*						
SI	0.54 (0.53–0.55)	0.52	30.11	85.69	10.6	95.6
ASI	0.56 (0.55–0.57)	50.63	42.05	71.77	7.7	95.6
MSI	0.54 (0.53–0.55)	0.67	25.85	88.96	11.7	95.5
AMSI	0.56 (0.55–0.58)	75.71	35.51	78.97	8.6	95.6

Abbreviations: AMSI, age-modified shock index; ASI, age-adjusted shock index; AUC, area under the curve; CI, confidence interval; MSI, modified shock index; MT, massive transfusion; SI, shock index.

**Table 3:** DeLong method-based statistical comparison of AUC.

	AUC difference	Standard error	95% confidence interval	*p* value
*MT*				
SI–ASI	0.00299	0.0107	−0.0181–0.0240	*p* = 0.7809
SI–MSI	0.00516	0.00166	0.00190–0.00842	*p* = 0.0019
SI–AMSI	0.00365	0.0108	−0.0176–0.0249	*p* = 0.7361
ASI–MSI	0.00815	0.0106	−0.0125–0.0288	*p* = 0.4399
ASI–AMSI	0.00664	0.00157	0.00356–0.00972	*p* < 0.0001
MSI–AMSI	0.00152	0.0104	−0.0190–0.0220	*p* = 0.8847

*In-hospital mortality*				
SI–ASI	0.109	0.0271	0.0555–0.162	*p* = 0.0001
SI–MSI	0.00217	0.0300	−0.0566–0.0609	*p* = 0.9423
SI–AMSI	0.115	0.0268	0.0628–0.168	*p* < 0.0001
ASI–MSI	0.106	0.00952	0.0878–0.125	*p* < 0.0001
ASI–AMSI	0.00680	0.00160	0.00366–0.00994	*p* < 0.0001
MSI–AMSI	0.113	0.00951	0.0946–0.132	*p* < 0.0001

*24 h mortality*				
SI–ASI	0.0176	0.0339	−0.0487–0.0840	*p* = 0.6022
SI–MSI	0.00311	0.00216	−0.00113–0.00735	*p* = 0.1505
SI–AMSI	0.023	0.0336	−0.0429–0.0889	*p* = 0.4941
ASI–MSI	0.0208	0.0338	−0.0454–0.0869	*p* = 0.5386
ASI–AMSI	0.00534	0.00197	0.00147–0.00922	*p* = 0.0068
MSI–AMSI	0.0261	0.0336	−0.0398–0.0920	*p* = 0.4376

Abbreviations: AMSI, age-modified shock index; ASI, age-adjusted shock index; AUC, area under the curve; MSI, modified shock index; MT, massive transfusion; SI, shock index.

## Data Availability

The data that support the findings of this study are available from the corresponding author upon reasonable request.
